# Supplementary figures and images for: Differential induction of interferon stimulated genes between type I and type III interferons is independent of interferon receptor abundance
Source: PLoS Pathog. 2018 Nov 28;14(11):e1007420. doi: 10.1371/journal.ppat.1007420 (PMC6287881; doi:10.1371/journal.ppat.1007420)

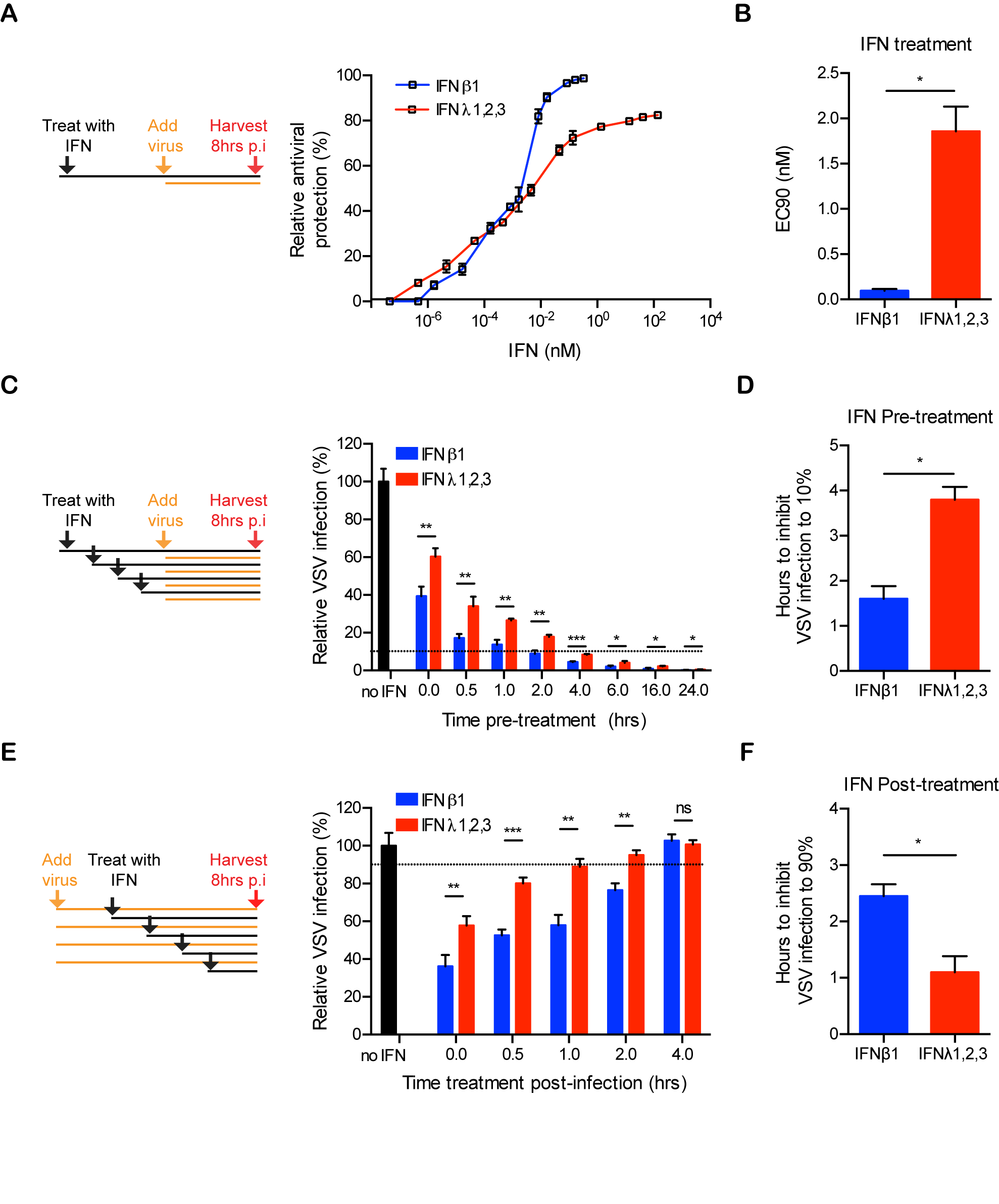

Supplement: S1 Fig — (A-B) Intestinal organoids were pre-treated with the indicated concentrations of type I IFN (β) or type III IFN (λ1−3) for 2.5 h prior to infection with VSV-Luc using a multiplicity of infection (MOI) of 1. Viral replication was assayed by measuring the luciferase activity. (A) The relative antiviral protection is expressed as a percentage of total protection in VSV-infected organoids or (B) as the EC90 corresponding to the concentration of type I IFN (β) or type III IFN (λ1−3) resulting in 90% inhibition (10% infection) of viral replication. (C-D) Intestinal organoids were treated with type I IFN (β) (2,000 RU/mL equivalent 0.33 nM) or type III IFN (λ1−3) (100ng/mL each or total 300 ng/mL equivalent 13.7 nM) for different times prior to infection with VSV-Luc. Viral replication was assayed by measuring luciferase activity. (C) The relative VSV infection is expressed as the percentage of the luciferase activity present in VSV-infected organoids without IFN treatment (set to 100). (D) Pre-incubation time of type I IFN (β) or type III IFN (λ1−3) required to inhibit VSV infection to 10% (90% inhibition). (E-F) Same as (C-D), except intestinal organoids were treated at the indicated times post-infection with VSV-Luc. (F) Delayed-time post-infection for type I IFN (β) or type III IFN (λ1−3) to still inhibit VSV infection to 90% (10% inhibition). Data represent the mean values of two independent experiments with intestinal organoids generated from two different donors. Error bars indicate the SD. *<P.05, **P < 0.01, ***P < 0.001, ns, not significant (unpaired t-test). (TIF) [file ppat.1007420.s001.tif]

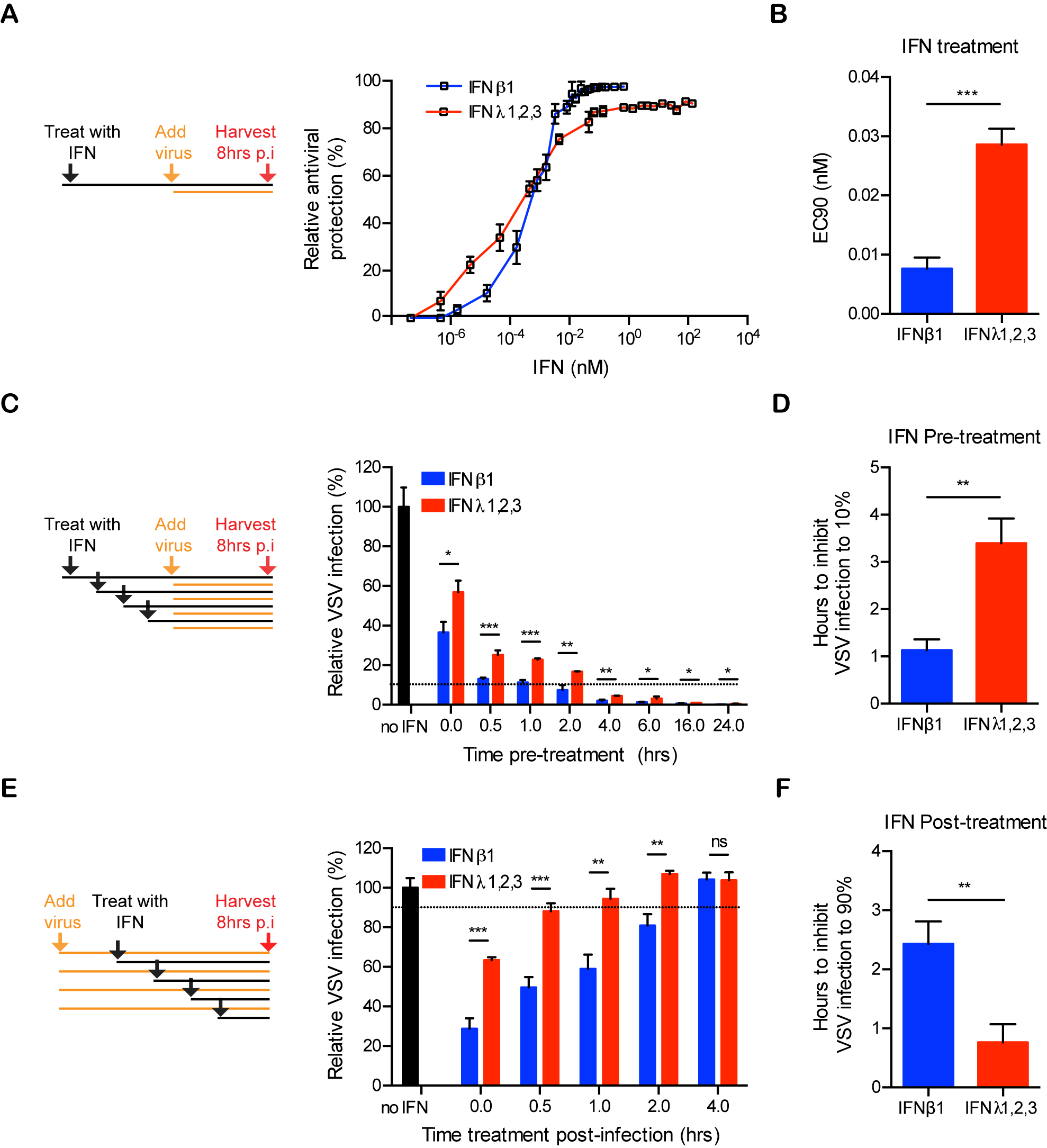

Supplement: S2 Fig — (A-B) T84 cells were pre-treated with the indicated concentrations of type I IFN (β) or type III IFN (λ1−3) for 2.5 h prior to infection with vesicular stomatitis virus (VSV) expressing Firefly luciferase (VSV-Luc) using a multiplicity of infection (MOI) of 1. Viral replication was assayed by measuring the luciferase activity. (A) The relative antiviral protection is expressed as a percentage of total protection in VSV-infected cells or (B) as the EC90 corresponding to the concentration of type I IFN (β) or type III IFN (λ1−3) resulting in 90% inhibition (10% infection) of viral replication. (C-D) T84 cells were treated with type I IFN (β) (2,000 RU/mL equivalent 0.33 nM) or type III IFN (λ1−3) (100ng/mL each or total 300 ng/mL equivalent 13.7 nM) for different times prior to infection with VSV-Luc. Viral replication was assayed by measuring luciferase activity. (C) The relative VSV infection is expressed as the percentage of the luciferase activity present in VSV-infected cells without IFN treatment (set to 100). (D) Pre-incubation time of type I IFN (β) or type III IFN (λ1−3) required to inhibit VSV infection to 10% (90% inhibition). (E-F) Same as (C-D), except T84 cells were treated at the indicated times post-infection with VSV-Luc. (F) Delayed-time post-infection for type I IFN (β) or type III IFN (λ1−3) to still inhibit VSV infection to 90% (10% inhibition). Data in (A–F) represent the mean values of three independent experiments. Error bars indicate the SD. *<P.05, **P < 0.01, ***P < 0.001, ****P < 0.0001, ns, not significant (unpaired t-test). (TIF) [file ppat.1007420.s002.tif]

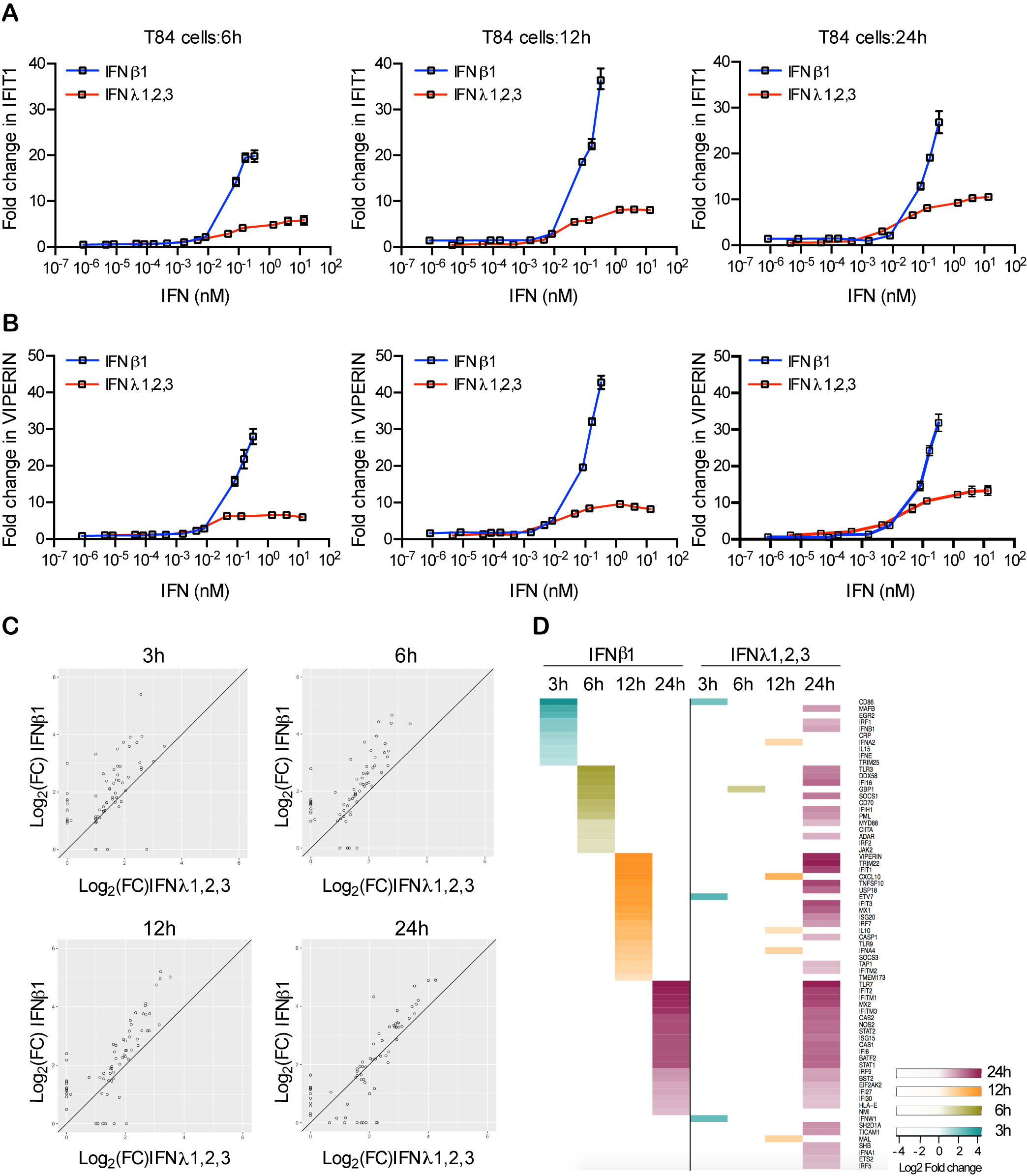

Supplement: S3 Fig — (A-B) T84 cells were stimulated with indicated concentrations of type I (β) or III IFN (λ1−3) for different times and the transcript levels of the ISGs IFIT1 and Viperin were analyzed by qRT-PCR. Data are normalized to TBP and HPRT1 and are expressed relative to untreated cells at each time point. A representative experiment with technical triplicates, out of three independent experiments is shown. Mean values and SD are shown. (C-D) T84 cells were treated with type I IFN (β) (2,000 RU/mL equivalent 0.33 nM) or type III IFN (λ1−3) (300 ng/mL equivalent 13.7 nM) for the indicated times and identification of the IFN-induced ISGs was performed by qRT-PCR. A total of 70 out of 132 ISGs tested were found to be significantly induced more than 2-fold compared with a baseline (mean of untreated controls at the particular time points) for at least one time point by at least one IFN treatment. Data are normalized to TBP and HPRT1. (C) Comparison of expression values (log2 (Fold Change)) for all genes induced at the indicated times with type I IFN (β) versus type III IFN (λ1−3). Solid line indicates equivalent expression. (D) Hierarchical clustering analysis of these genes produced four distinct temporal expression patterns (Groups 1–4) based on the time-point of the maximum induction in response to type I IFN (β) or type III IFN (λ1−3). Color codes have been used to visualize the induction peak per group. Gene expression heat map showing the genes clustered in their respective temporal expression patterns groups in response to type I IFN (β) or type III IFN (λ1−3). The genes per group are sorted in decreasing order on the basis of their fold change of expression in response to type I IFN (β) or type III IFN (λ1−3) and only showing the highest expressed values within the temporal groups omitting all other values for visualization. (TIF) [file ppat.1007420.s003.tif]

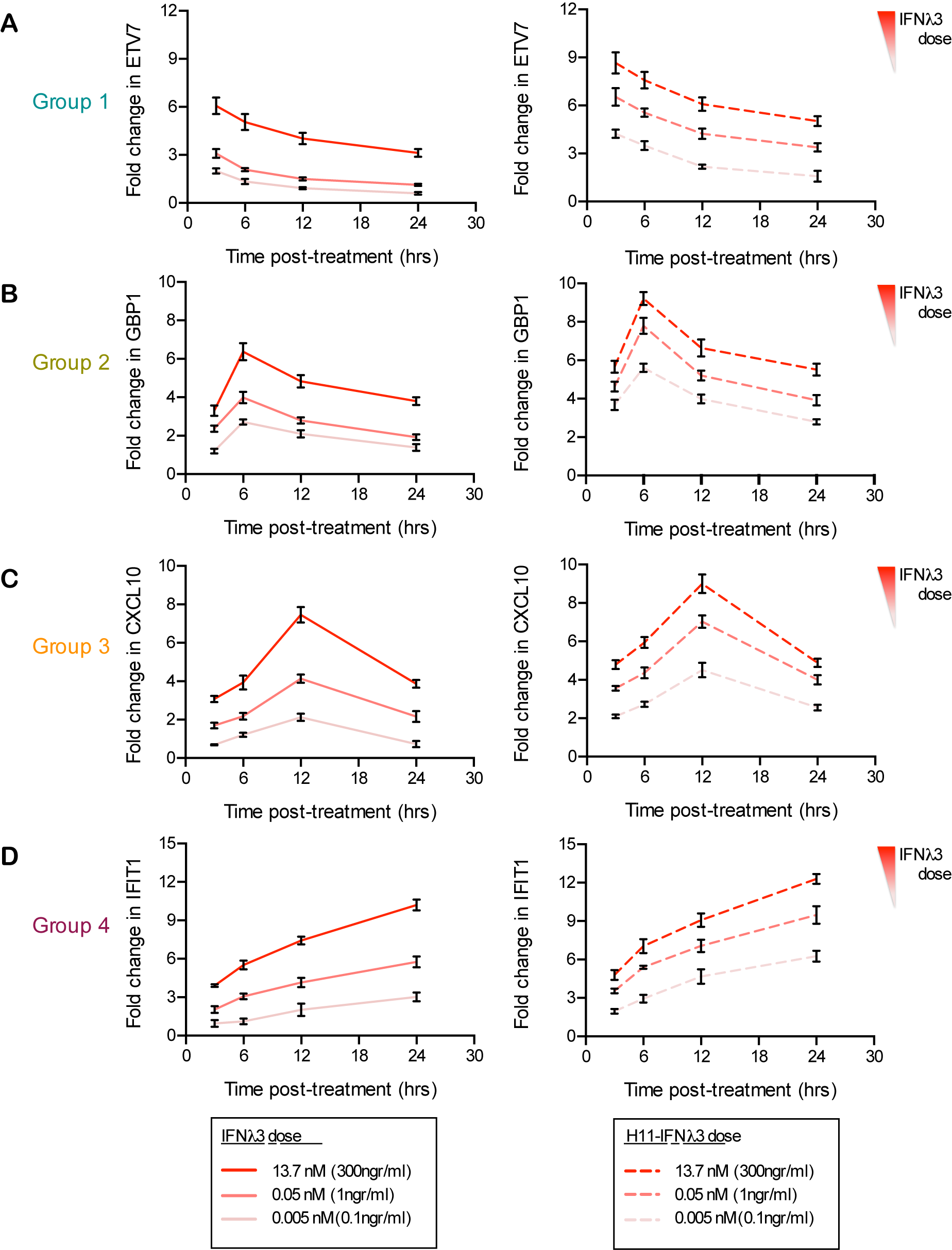

Supplement: S4 Fig — (A-D) T84 cells were stimulated with increasing concentrations of type III IFN (λ3) (WT-IFNλ3) or the high affinity IFNλ3 variant (H11-IFNλ3) for indicated times and the kinetic of expression of one representative ISG from each temporal expression groups 1–4 was analyzed by qRT-PCR, (left column) WT-IFNλ3, (right column) H11-IFNλ3 treated cells. Data are normalized to HPRT1 and are expressed relative to untreated cells at each time point. A representative experiment with technical triplicates. Mean values and SD are shown. (TIF) [file ppat.1007420.s004.tif]

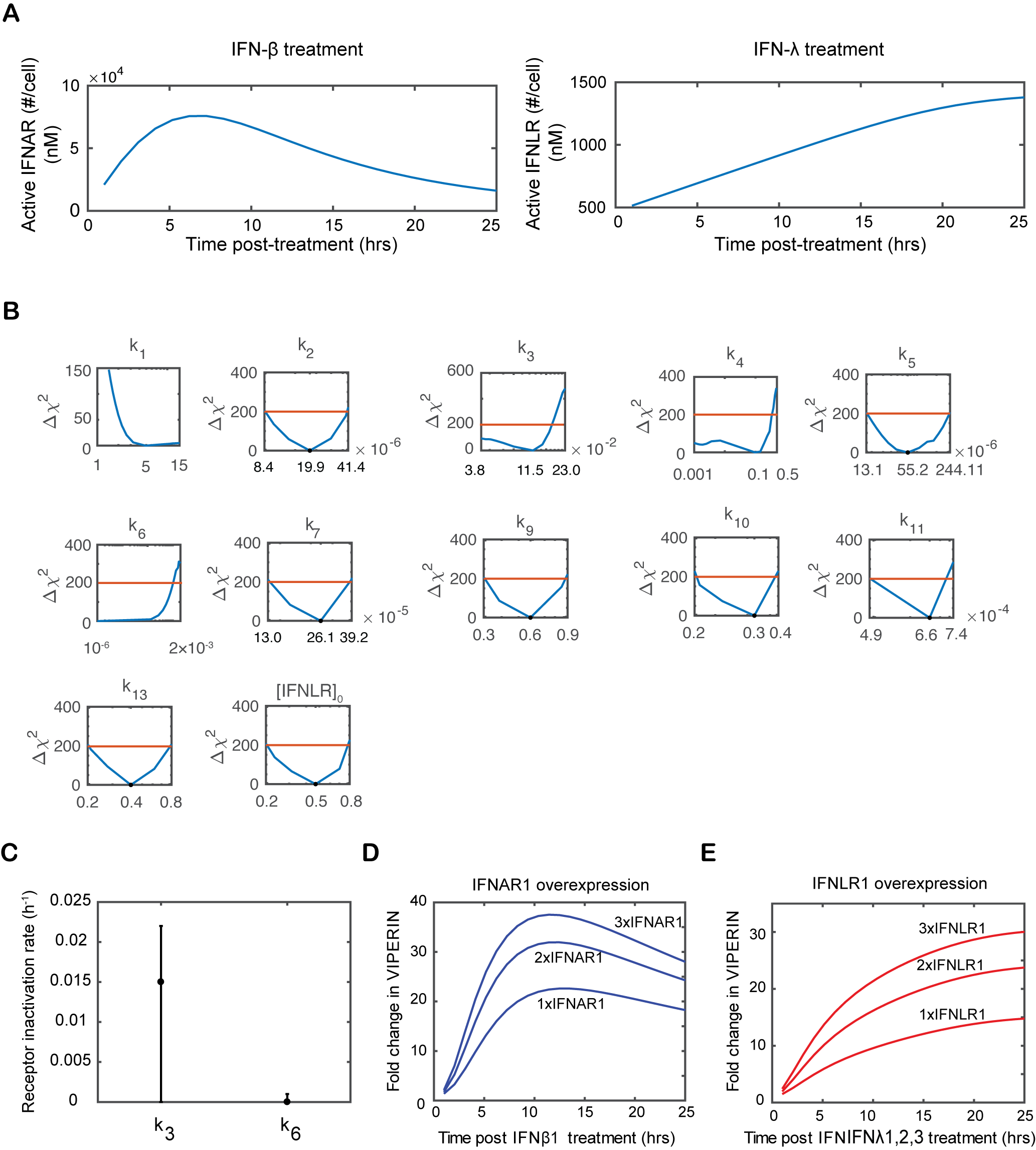

Supplement: S5 Fig — (A) Comparative simulation of type I and type III IFN receptor complex activation. Cellular concentration of the activated type I or type III IFN receptor complex, upon treatment with 0.1 nM of IFNs, are simulated using the calibrated model. (B) Profile likelihoods of model parameters. The uncertainty of the estimated model parameters is calculated using the profile likelihood method. The solid blue line is the change in the weighted sum of squared residuals (Δχ2), the filled circle indicates the optimum parameter value, and the solid red line indicates the 95% threshold calculated using the χ2 distribution. (C) The 95% confidence bounds of type I or type III IFN receptor complex inactivation rate constants are calculated using the profile likelihood method. Our calculations show that the type III IFN receptor complex inactivation rate constant (k2) is significantly smaller than the corresponding rate constant for type I IFN receptor complex (k11). (D-E) The mathematical model shows the effect of IFNAR1 (D) and IFNLR1 (E) overexpression of up to 3-fold (3×IFNAR1 and 3×IFNLR1) on Viperin activation upon treatment with representative concentrations of type I IFN (β) (0.1 nM) or type III IFN (λ1−3) (13.7 nM), respectively. (TIF) [file ppat.1007420.s005.tif]

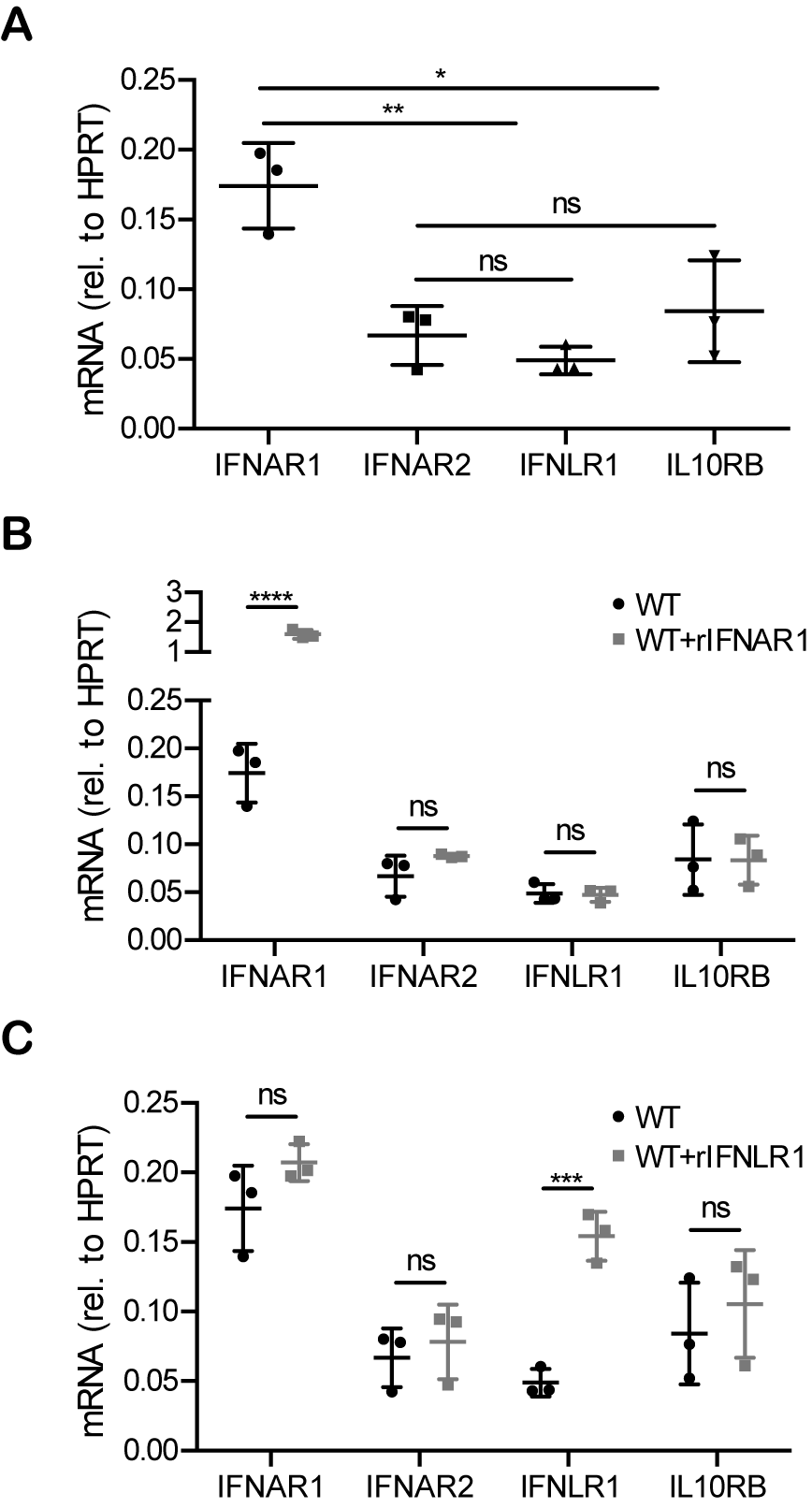

Supplement: S6 Fig — (A) T84 wild-type cells, (B) T84 cells overexpressing rIFNAR1 (WT+rIFNAR1) and (C) T84 cells overexpressing rIFNLR1 (WT+rIFNLR1) were analyzed by qRT-PCR to quantify the transcript levels of IFNAR1, IFNAR2, IFNLR1 and IL10RB (IFNLR2). Data are normalized to HPRT1. The mean value obtained from three independent experiments is shown. Error bars indicate the SD. *<P.05, **P < 0.01, ***P < 0.001, ****P < 0.0001, ns, not significant (unpaired t-test). (TIF) [file ppat.1007420.s006.tif]

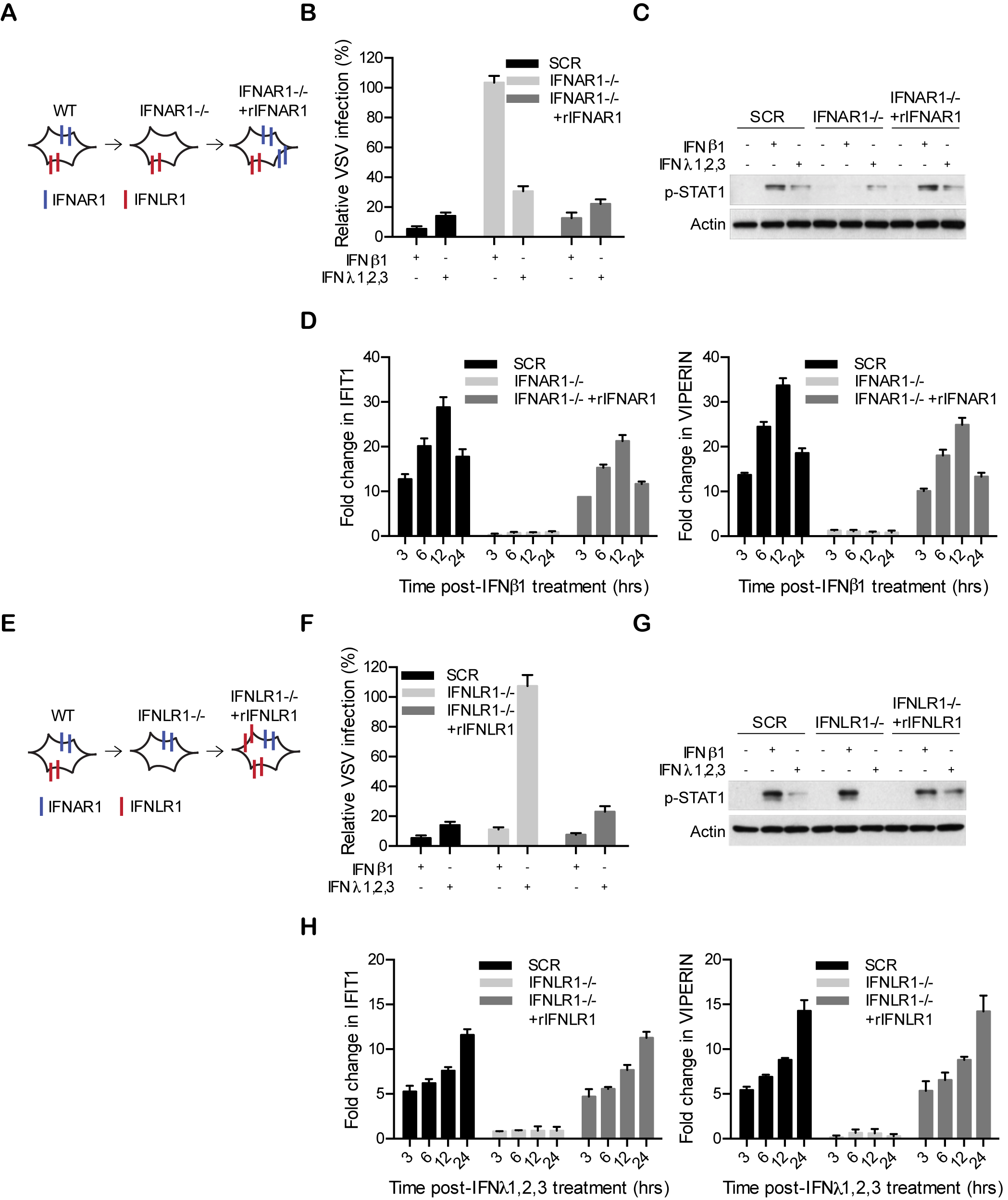

Supplement: S7 Fig — (A-D) T84 IFNAR1-/- cells were rescued by stable expression of a cleavage resistant mutant of rIFNAR1 (see methods for details) (T84 IFNAR1-/- + rIFNAR1). (B-D) T84 IFNAR1-/-, T84 IFNAR1-/- + rIFNAR1 cells and control T84 cells scramble gRNA (SCR) were pre-treated with type I IFN (β) (2,000 RU/mL equivalent 0.33 nM) or type III IFN (λ1−3) (300 ng/mL equivalent 13.7 nM). (B) 2.5 h post-treatment, T84 cells were infected with VSV-Luc (MOI = 1). Viral replication was assayed by measuring the luciferase activity. For each sample luciferase activity was measured in triplicates and is expressed as the percentage of the luciferase signal in VSV-infected cells without IFN treatment (set to 100) for each cell lines. (C) 1h post IFN treatment, IFN signaling was measured by immunoblotting for pSTAT1 Y701. Actin was used as a loading control. A representative immunoblot out of three independent experiments is shown. (D) same as (B), except that induction of IFN-stimulated genes was monitored by relative qRT-PCR quantification of IFIT1 and Viperin at the indicated times post-IFN treatment. (E-H) same as (A-D) except that T84 IFNLR1-/- were rescued by stable expression of a cleavage resistant mutant of rIFNLR1 (T84 IFNLR1-/- + rIFNLR1). Data were normalized to HPRT1 and are expressed relative to untreated cells of each time point. The mean value obtained from three independent experiments is shown. Error bars indicate the SD. (TIF) [file ppat.1007420.s007.tif]

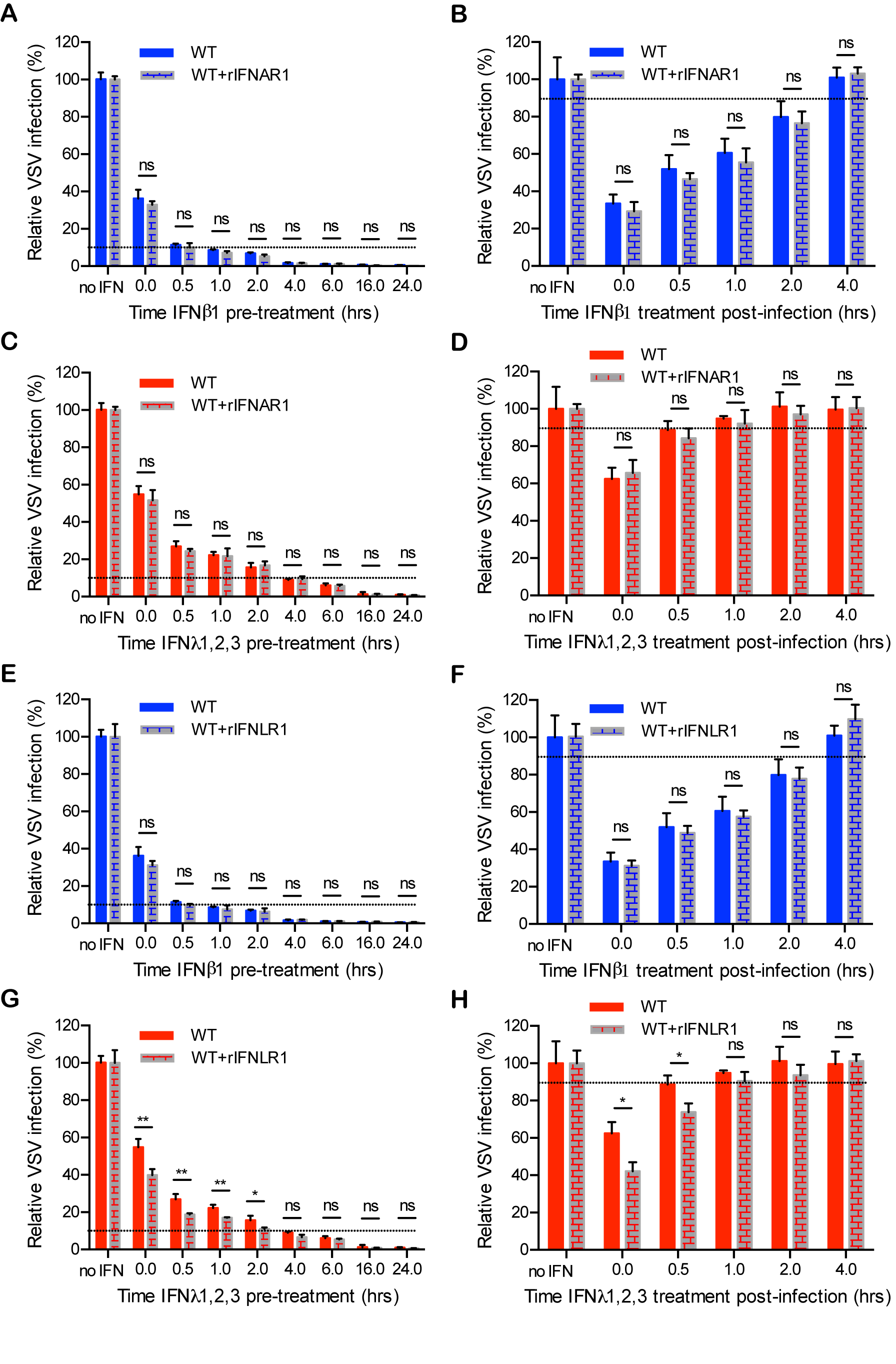

Supplement: S8 Fig — (A-H) Wild-type T84 cells were transduced with rIFNAR1 or rIFNLR1 to create stable lines overexpressing either receptor. (A-D) T84 wild-type cells (WT) and T84 cells overexpressing the IFNAR1 (WT+rIFNAR1) were treated with (A-B) type I IFN (β) (2,000 RU/mL equivalent 0.33 nM) or (C-D) type III IFN (λ1−3) (100ng/mL each = 300 ng/mL equivalent 13.7 nM) at the indicated times (A, C) prior to infection or (B, D) post infection with VSV-Luc. Viral replication was assayed by measuring the luciferase activity. The time necessary to confer IECs an antiviral state was addressed by measuring the impact of IFN treatment on viral replication. For each sample luciferase activity was measured in triplicates and is expressed relative to VSV-infected cells without IFN treatment (set to 100). (E-H) same as (A-D), but T84 wild-type cells (WT) and T84 cells overexpressing the IFNLR1 (WT+rIFNLR1) were treated with (E-F) type I IFN (β) (2,000 RU/mL equivalent 0.33 nM) or (G-H) type III IFN (λ1−3) (100ng/mL each = 300 ng/mL equivalent 13.7 nM) at the indicated times (E, G) prior to infection or (F, H) post infection with VSV-Luc. Data represent the mean values of three independent experiments. Error bars indicate the SD. *<P.05, **P < 0.01, ns, not significant (unpaired t-test). (TIF) [file ppat.1007420.s008.tif]

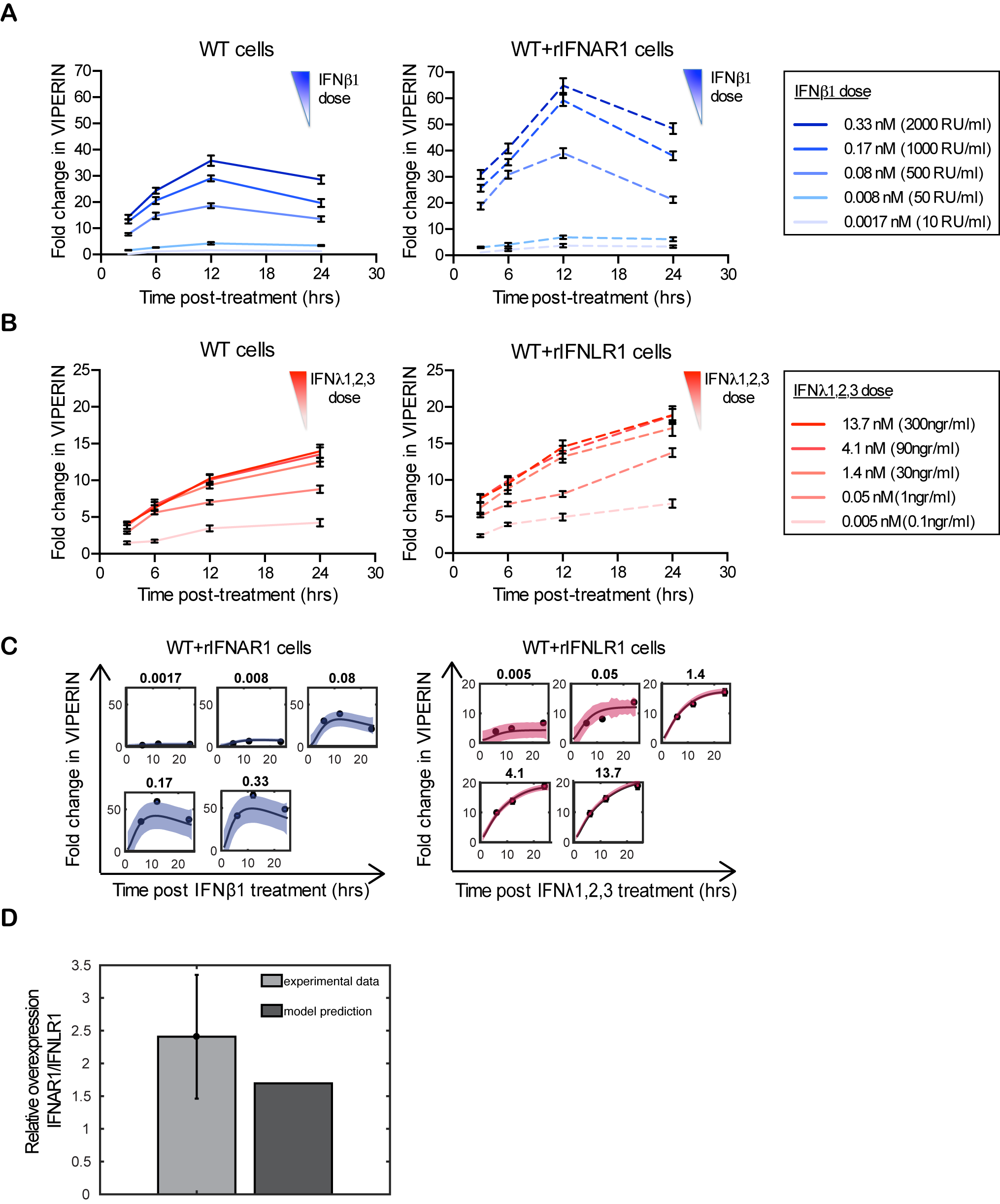

Supplement: S9 Fig — (A-B) Wild-type T84 cells were transduced with rIFNAR1 or rIFNLR1 to create stable lines overexpressing either receptors. (A) T84 wild-type cells (WT) and T84 cells overexpressing the IFNAR1 (WT+rIFNAR1) were treated with increasing concentrations of type I IFN (β) for the indicated times and the expression kinetics of the ISG Viperin were analyzed by qRT-PCR. Data are normalized to HPRT1 and are expressed relative to untreated cells at each time point. (B) Same as (A), except T84 cells overexpressing the IFNLR1 (WT+IFNLR1) were used and treated with increasing concentrations of type III IFN (λ1−3). A representative experiment with technical triplicates, out of three independent experiments is shown. Mean values and SD are shown. (C-D) The mathematical model predicts the effect of IFNAR1 and IFNLR1 overexpression on Viperin activation upon treatment with different concentrations of type I IFN (β) and type III IFN (λ1−3). The IFNAR1 and IFNLR1 levels were increased ~2.6 and ~1.5 fold, respectively, while all other parameters were held constant. The solid lines are the best fits and the shaded areas are 95% confidence intervals. (D) The mathematical model correctly predicts the IFNAR1 versus IFNLR1 overexpression, measured experimentally by qRT-PCR. (TIF) [file ppat.1007420.s009.tif]

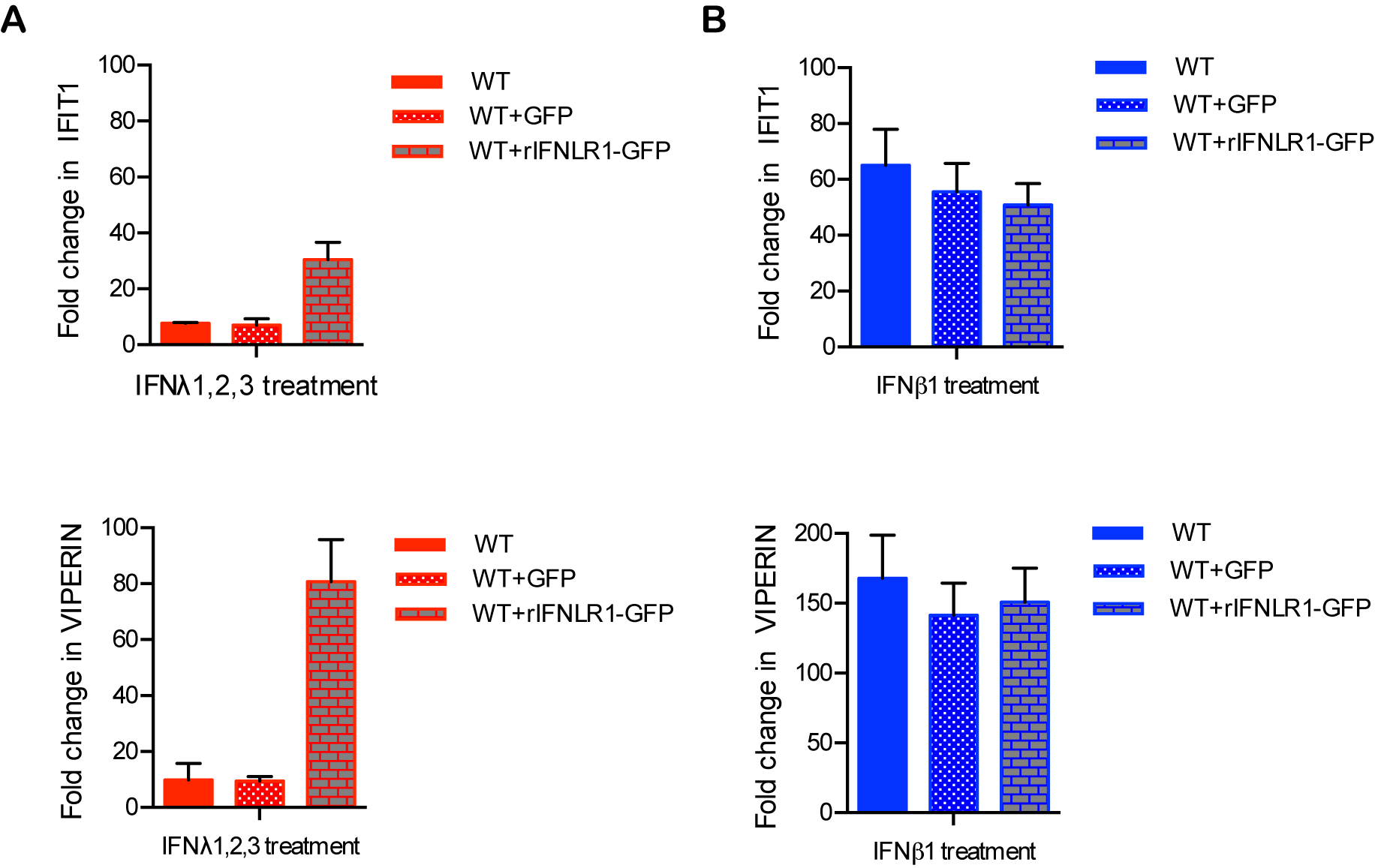

Supplement: S10 Fig — Wild-type 293 HEK cells were transfected with rIFNLR1-GFP or GFP alone. 293 HEK wild-type cells (WT), 293 HEK cells overexpressing the rIFNLR1-GFP (WT+rIFNLR1-GFP) or GFP alone (WT+GFP) were treated with (A) type III IFN (λ1−3) (300 ng/mL equivalent 13.7 nM) or (B) type I IFN (β) (2,000 RU/mL equivalent 0.33 nM) for 12 hours and the transcript levels of the ISGs IFIT1 and Viperin were analyzed by qRT-PCR. Data are normalized to HPRT1 and are expressed relative to untreated cells. Data represent the mean values of two independent experiments. Mean values and SD are shown. (TIFF) [file ppat.1007420.s010.tiff]
