# Supplementary material for: Differential induction of interferon stimulated genes between type I and type III interferons is independent of interferon receptor abundance
Source: PLoS Pathog. 2018 Nov 28;14(11):e1007420. doi: 10.1371/journal.ppat.1007420 (PMC6287881; doi:10.1371/journal.ppat.1007420)
Supplement: S1 Table — For the gene expression analysis of interferon stimulated genes (ISGs), qRT-PCR was performed using a predesigned 384-well assay of type I IFN response assaying the expression of ISGs. The Reference Sequence (RefSeq) accession number is provided for each ISG tested. (PDF) [file ppat.1007420.s011.pdf]

S1 Table. List of primers used in predesigned 384 well assay qRT-PCR.

	Gene Symbol	RefSeq Accession No		Gene Symbol	RefSeq Accession No
#1	ADAR	NC_000001.10, NG_011844.1, NT_004487.19	#45	IL15	NC_000004.11, NT_016354.19
#2	B2M	NC_000015.9, NG_012920.1, NT_010194.17	#46	IL6	NC_000007.13, NG_011640.1, NT_007819.17
#3	BAG3	NC_000010.10, NG_016125.1, NT_030059.13	#47	IRF1	NC_000005.9, NG_011450.1, NT_034772.6
#4	BST2	NC_000019.9, NT_011295.11	#48	IRF2	NC_000004.11, NT_016354.19
#5	CASP1	NC_000011.9, NT_033899.8	#49	IRF3	NC_000019.9, NT_011109.16
#6	CAV1	NC_000007.13, NT_007933.15, NG_012051.1	#50	IRF5	NC_000007.13, NT_007933.15, NG_012306.1
#7	CCL2	NC_000017.10, NG_012123.1, NT_010799.15	#51	IRF7	NC_000011.9, NT_009237.18
#8	CCL5	NC_000017.10, NG_015990.1, NT_010799.15	#52	IRF9	NC_000014.8, NT_026437.12
#9	CD70	NC_000019.9, NT_011255.14	#53	ISG15	NC_000001.10, NT_004350.19
#10	CD80	NC_000003.11, NT_005612.16	#54	ISG20	NC_000015.9, NT_010274.17
#11	CD86	NC_000003.11, NT_005612.16	#55	JAK1	NC_000001.10, NG_023402.1, NT_032977.9
#12	CDKN1B	NC_000012.11, NG_016341.1, NT_009714.17	#56	JAK2	NC_000009.11, NG_009904.1, NT_008413.18
#13	CIITA	NC_000016.9, NG_009628.1, NT_010393.16	#57	MAL	NC_000002.11, NT_022171.15
#14	CRP	NC_000001.10, NG_013007.1, NT_004487.19	#58	MET	NC_000007.13, NT_007933.15, NG_008996.1
#15	CXCL10	NC_000004.11, NT_016354.19	#59	MNDA	NC_000001.10, NT_004487.19
#16	DDX58	NC_000009.11, NT_008413.18	#60	MX1	NC_000021.8, NG_027788.1, NT_011512.11
#17	EIF2AK2	NC_000002.11, NT_022184.15	#61	MX2	NC_000021.8, NT_011512.11
#18	GBP1	NC_000001.10, NT_032977.9	#62	MYD88	NC_000003.11, NG_023225.1, NT_022517.18, NG_016964.1
#19	GUSB	NC_000007.13, NG_016197.1, NT_007933.15	#63	NMI	NC_000002.11, NT_005403.17
#20	HLA-A	NC_000006.11, NT_007592.15, NT_113891.2, NT_167244.1	#64	NOS2	NC_000017.10, NG_011470.1, NT_010799.15
#21	HLA-B	NC_000006.11, NG_002397.2, NG_023187.1, NT_007592.15, NT_113891.2,	#65	OAS1	NC_000012.11, NT_009775.17, NG_011530.1
		NT_167246.1, NT_167247.1, NT_167248.1, NT_167249.1	#66	OAS2	NC_000012.11, NT_009775.17
#22	HLA-E	NC_000006.11, NT_007592.15, NT_113891.2, NT_167245.1, NT_167246.1,	#67	PML	NC_000015.9, NT_010194.17
		NT_167247.1, NT_167248.1, NT_167249.1	#68	PRKCZ	NC_000001.10, NT_004350.19
#23	HLA-G	NC_000006.11, NT_007592.15, NT_113891.2, NT_167245.1, NT_167246.1,	#69	PSME2	NC_000014.8, NT_026437.12
		NT_167247.1, NT_167248.1, NT_167249.1	#70	RPLP0	NC_000012.11, NT_009775.17
#24	HPRT1	NC_000023.10, NG_012329.1, NT_011786.16	#71	SH2D1A	NC_000023.10, NT_011786.16, NG_007464.1
#25	IFI16	NC_000001.10, NT_004487.19	#72	SHB	NC_000009.11, NT_008413.18
#26	IFI27	NC_000014.8, NT_026437.12	#73	SOCS1	NC_000016.9, NT_010393.16
#27	IFI30	NC_000019.9, NT_011295.11	#74	STAT1	NC_000002.11, NG_008294.1, NT_005403.17
#28	IFI6	NC_000001.10, NT_004610.19	#75	STAT2	NC_000012.11, NT_029419.12
#29	IFIH1	NC_000002.11, NG_011495.1, NT_005403.17	#76	STAT3	NC_000017.10, NT_010783.15, NG_007370.1
#30	IFIT1	NC_000010.10, NT_030059.13	#77	TAP1	NC_000006.11, NG_011759.1, NG_028165.1, NT_007592.15, NT_113891.2, NT_167244.1,
#31	IFIT2	NC_000010.10, NT_030059.13			NT_167245.1, NT_167246.1, NT_167247.1, NT_167248.1, NT_167249.1
#32	IFIT3	NC_000010.10, NT_030059.13	#78	TBP1	NC_000006.11, NT_025741.15, NG_008165.1
#33	IFITM1	NC_000011.9, NT_009237.18	#79	TICAM1	NC_000019.9, NT_011255.14
#34	IFITM2	NC_000011.9, NT_009237.18	#80	TIMP1	NC_000023.10, NG_008437.1, NG_012533.1, NT_079573.4
#35	IFITM3	NC_000011.9, NT_009237.18	#81	TLR3	NC_000004.11, NG_007278.1, NT_016354.19
#36	IFNA1	NC_000009.11, NT_008413.18	#82	TLR7	NC_000023.10, NG_012569.1, NT_167197.1
#37	IFNA2	NC_000009.11, NT_008413.18	#83	TLR8	NC_000023.10, NG_012882.1, NT_167197.1
#38	IFNA4	NC_000009.11, NT_008413.18	#84	TLR9	NC_000003.11, NT_022517.18
#39	IFNAR1	NC_000021.8, NT_011512.11	#85	TMEM1	NC_000005.9, NT_029289.11

				73	
#40	IFNAR2	NC_000021.8, NG_012089.1, NT_011512.11, NG_016003.1	#86	TNFSF10	NC_000003.11, NT_005612.16
#41	IFNB1	NC_000009.11, NT_008413.18	#87	TRAF3	NC_000014.8, NT_026437.12, NG_027973.1
#42	IFNE	NC_000009.11, NT_008413.18	#88	TYK2	NC_000019.9, NG_007872.1, NT_011295.11
#43	IFNW1	NC_000009.11, NT_008413.18	#89	VEGFA	NC_000006.11, NG_008732.1, NT_007592.15
#44	IL10	NC_000001.10, NG_012088.1, NT_167186.1			
