# Supplementary material for: Differential induction of interferon stimulated genes between type I and type III interferons is independent of interferon receptor abundance
Source: PLoS Pathog. 2018 Nov 28;14(11):e1007420. doi: 10.1371/journal.ppat.1007420 (PMC6287881; doi:10.1371/journal.ppat.1007420)
Supplement: S2 Table — The expression of additional ISGs, transcriptional factors and housekeeping genes was analyzed by qRT-PCR with the primer sets shown in this table. (PDF) [file ppat.1007420.s012.pdf]

S2 Table. List of primer sequences used for qRT-PCR analysis.

	Gene Symbol	Forward primer (5’-3’)	Reverse primer (5’-3’)
#1	ARID3A	CCACGGCGACTGGACTTA	GCTGAACAAGTCATCCAGGAAT
#2	BLZF1	CATTAACAAATCACAAGTTGGCAAA	TGCAGAATTCAACTGTTGATGG
#3	CBFB	GCGAGTGTGAGATTAAGT	AGAGACAGATTGGTTCCT
#4	CEBPD	ACTTACCACCACTAAACTGC	TGTACCTTAGCTGCATCAAC
#5	CREB3L3	TTTAGCTGCTGGAAAGATGG	TCCACGTGTCTCAGGATG
#6	CSDA	TTCTCGCCACCAAAGTCCTTG	TTCTTCTTGATGGCAGTCTGATGT
#7	CXCL10	TGAAATTATTCCTGCAAGCCAA	GACATCTCTTCTCACCCTTCTTT
#8	DRAP1	ACATCCCACCTGAAGCAG	ATCCATGTGGTTGTCTTCC
#9	EGR1	CAGCACCTTCAACCCTCAG	AGCGGCCAGTATAGGTGATG
#10	EGR2	GCTGACACGGCACATCC	ACAGTAGTCACAGGCGAAGG
#11	ELF1	TGTCATGCTGCAGTCACAAA	ACTGCGAGGAGAAAAGGTCA
#12	ELK4	CTGGTGCCAAGACCTCTAGC	TCGGCTGGATTCTCAGTCTT
#13	ETS2	TCTGCCTCAATAAGCCAACC	TAAACTCCCATCCGTCTCCA
#14	ETV6	AAGCCCATCAACCTCTCTCA	CCATCGGATGAAGTTTTCGT
#15	ETV7	AAGAACCGGGTGAACATGAC	TTGTCCTGGACCATCTTTCC
#16	EWSR1	TAGGATATGGACAGAGTAAC	GTAGAGGAATAGCTGGTAG
#17	FUBP1	ACGCTTTCAAAGATGCACTG	TTTTTGTCCCCCATAACCAT
#18	GBP1	CTATGAGGAACCGAG	CACGTTCCACTTCAATCTCC
#19	HDAC2	AGCATCAGGATTCTGTTACGTTAATGA	CAACACCATCACCATGATGAATATCT
#20	HIF1A	GCTATTTGCGTGTGAGGAAAC	CACCATCATCTGTGAGAACCA
#21	HPRT1	CCTGGCGTCGTGATTAGTGAT	AGACGTTCAGTCCTGTCCATAA
#22	IFIT1	AAAAGCCCACATTTGAGGTG	GAAATTCCTGAAACCGACCA
#23	IFITM3	GATGTGGATCACGGTGGAC	AGATGCTCAAGGAGGAGCAC
#24	IFNAR1	CACTGACTGTATATTGTGTGAAAGCCA GAG	CATCTATACTGGAAGAAGGTTTAAGTGATG
#25	IFNAR2	ATTTCCGGTCCATCTTATCAT	ACTGAACAACGTTGTGTTCC
#26	IL10RB	TTGCTGTGGTGCGTTTACAAG	CTTTCAGGTGCTGTGGAAGAGA
#27	IFNLR1	ACCTATTTTGTGGCCTATCAGAGC	CGGCTCCACTTCAAAAAGGTAAT
#28	IRF1	CCAAGAGGAAGTCATGTG	TAGCCTGGAACTGTGTAG
#29	IRF4	ATCACAGCTCACGTAGAA	ATAGAGGAATGGCGGATA
#30	IRF8	GGCATTCTCGGAGGAGTAGA	GTTACAGCATCCAGGCCATC
#31	JUN	GAGGACCGGAGACAAGTG	CCTTCTTCTCTTGCGTGG
#32	JUNB	CATACACAGCTACGGGATAC	TTTGAGACTCCGGTAGGG
#33	MAFB	GACGCAGCTCATTCAGCAG	CCGGAGTTGGCGAGTTTCT
#34	MAFF	GATTGAGAGATACAGAGCCG	CTAGCTTTGAATCCTGGGAG
#35	MAX	AACCGAGGTTTCAATCTGC	GGGATGCCTTCTCTCCTTG
#36	MYC	GATTCTCTGCTCTCCTCGAC	CTTGTTCCTCCTCAGAGTC
#37	MXA	GAGCTGTTCTCCTGCACCTC	CTCCCACTCCCTGAAATCTG
#38	NFIL3	AATGCAGACCGTCAAAAAGG	AGCAGCTCCTCACCTGTTGT

#39	NFE2	AGCTGCAGGGTCTGAATG	CAGAATCTGGGTGGATTGAG
#40	NPAS2	AGGAGCTGGCTCTGGAAGAC	CCGAGGTTCCAGGCTTGAG
#41	RASD1	CAAGACCGGGGAGAATACCTG	GCGAGAATGTCCAAATACTCACC
#42	RELA	TATCAGTCAGCGCATCCAGACCAA	AGAGTTTCGGTTCACTCGGCAGAT
#43	SMAD3	AGCAATATTCCAGAGACC	GCAGGTCCAAGTTATTATG
#44	SMAD4	TTACCATCATAACAGCACTA	CAAGCTCATTGTGAACAG
#45	SOCS3	GCGAGGATCCTGGTGACA	CCAGGATGGTTCCCTTCAG
#46	SREBF1	ACTTCTGGAGGCATCGCAAGCA	AGGTTCCAGAGGAGGCTACAAG
#47	TBP	TGCACAGGAGCCAAGAGTGAA	CACATCACAGCTCCCCACCA
#48	TCF7L2	CATATGGTCCCACCACATCA	CACTCTGGGACGATTCCTGT
#49	TEAD4	ATGATCATCACCTGCTCCAC	GTCCATTCTCATAGCGAGCA
#50	TLR3	TTGTCTTCTGCACGAACCTGCGC	AACGCAAGGATTTTATT
#51	TRIM21	TGGAGACCTTTAGGGGGTTT	TGAGCGGAAACTGAAAGTGA
#52	TRIM22	GGATGCCAGCACGCTCATCTCAG	TTCAGCATCACGTCCACCCAGTAGT
#53	TRIM25	AACATCTCTCAAGGCCAAGGT	AGATGCCTACCCCACAGAAGT
#54	USP18	ATGTGAGCCAGGCACGAT	TCCCGACGTGGAACTCAG
#55	VIPERIN	GAGAGCCATTTCTTCAAGACC	CTATAATCCCTACACCACCTCC
#56	ZFP36L2	CTTGATAGTTAGCCCTCAGC	CCAAGTAACCAGTATGGACC
