# Supplementary material for: Differential induction of interferon stimulated genes between type I and type III interferons is independent of interferon receptor abundance
Source: PLoS Pathog. 2018 Nov 28;14(11):e1007420. doi: 10.1371/journal.ppat.1007420 (PMC6287881; doi:10.1371/journal.ppat.1007420)
Supplement: S3 Table — The table lists all differential equations explaining the dynamics of different biological species in our model. Cell surface (Area) is calculated as, Area = (36∙π)1/3∙Vcell2/3. Cell volume (Vcell) is assumed equal to 2×10^−9^ Liter. Brackets [] indicate the concentration of the respective biological species. (PDF) [file ppat.1007420.s013.pdf]

S3 Table. Mathematical formulation of the models. Models are implemented as system of ordinary differential equations. Alternative models can be generated by using the same or different rates for different biological processes. These alternative rates are shown inside brackets like {k_x, k_y}. Square brackets indicate concentration [ ].

ODEs model biological system dynamics	Explanation
$\frac{d[IFNAR]}{dt} = k_1 \cdot [IFNAR]_0 - \left(k_2 \cdot [IFN_\beta] \cdot [IFNAR] + k_1 \cdot [IFNAR]\right)$	IFNAR dynamics
$\frac{d[IFNAR^*]}{dt} = k_2 \cdot [IFN_\beta] \cdot [IFNAR] - k_3 \cdot [IFNAR^*] \cdot [ISG]$	Activated IFNAR dynamics
$\frac{d[IFNLR]}{dt} = k_4 \cdot [IFNLR]_0 - (\{k_2, k_5\} \cdot [IFN_\lambda] \cdot [IFNLR] + k_4 \cdot [IFNLR])$	IFNLR dynamics
$\frac{d[IFNLR^*]}{dt} = \{k_2, k_5\} \cdot [IFN_\lambda] \cdot [IFNLR] - \{k_3, k_6\} \cdot [IFNLR^*] \cdot [ISG]$	Activated IFNLR dynamics
$\frac{d[pSTAT]}{dt} = \{k_7, k_8\} \cdot ([IFNAR^*] + [IFNLR^*]) \cdot ([STAT]_{tot} - [pSTAT]) - k_9 \cdot [pSTAT]$	Active STAT1/2 dynamics
$\frac{d[ISG]}{dt} = k_{10} \cdot [ISG]_0 + \{k_{11}, k_{12}\} \cdot [pSTAT] - k_{10} \cdot [ISG]$	ISG activation dynamics (Viperin)
$\frac{d[IFN_\beta]}{dt} = -k_{13} \cdot [IFN_\beta]$	IFN-β dynamics
$\frac{d[IFN_\lambda]}{dt} = -k_{13} \cdot [IFN_\lambda]$	IFN-λ dynamics

The best ranked model, model M_5, has different rates of receptor activation and inactivation, uses k_2, k_3, k_5 and k_6 while lacking other sources of difference, namely k_8 and k_{12}.
