# Supplementary material for: Differential induction of interferon stimulated genes between type I and type III interferons is independent of interferon receptor abundance
Source: PLoS Pathog. 2018 Nov 28;14(11):e1007420. doi: 10.1371/journal.ppat.1007420 (PMC6287881; doi:10.1371/journal.ppat.1007420)
Supplement: S4 Table — Biological species considered in the model (state variables) and their initial values are listed in the table. (PDF) [file ppat.1007420.s014.pdf]

S4 Table. State variables and initial values. The concentrations are converted to number of molecules per nanoliter ($\#\cdot nL^{-1}$). Square brackets indicate concentration [ ].

State variable	Initial value	Explanation
[IFN-β] ($\#\cdot nL^{-1}$)	(8.30×10^{-7}-0.33 nM)$\times$***f2nM***	Interferon beta
[IFN-λ] ($\#\cdot nL^{-1}$)	(4.56×10^{-6}-13.70 nM) $\times$***f2nM***	Interferon lambda
[IFNAR] ($\#\cdot nL^{-1}$)	500	Cellular IFNAR concentration[1]
[$IFNAR^*$] ($\#\cdot nL^{-1}$)	0	Activated IFNAR concentration
[IFNLR] ($\#\cdot nL^{-1}$)	250	Cellular IFNLR concentration (Estimated)
[$IFNLR^*$] ($\#\cdot nL^{-1}$)	0	Activated IFNLR
$[STAT]_{tot}$ ($\#\cdot nL^{-1}$)	32000	Total STAT1 [2]
[pSTAT] ($\#\cdot nL^{-1}$)	0	Active STAT1/2 dimer
[ISG] ($\#\cdot nL^{-1}$)	1	Interferon stimulated Gene[3]
f2nM	$10^{-18}\times$***Avogadro number***	Factor converting nM to $\#\cdot nL^{-1}$

1-The initial value is calculated assuming 10^3 IFNAR molecule per cell. The human epithelial cell volume is considered 2 nanoliter (How big is a cell?, cell biology by the numbers).

2- Total STAT1 per nL is calculated according to the STAT1 counts per cell [72].

3- It is assumed that both Viperin and the control gene has 2 copy numbers per cell.
