# Supplementary material for: Differential induction of interferon stimulated genes between type I and type III interferons is independent of interferon receptor abundance
Source: PLoS Pathog. 2018 Nov 28;14(11):e1007420. doi: 10.1371/journal.ppat.1007420 (PMC6287881; doi:10.1371/journal.ppat.1007420)
Supplement: S5 Table — The estimated value of the model free parameters, their profile-likelihood based confidence bound and their dimensions are explained in the table. All reaction rate constants of the model, k1-k9, are practically identifiable. (PDF) [file ppat.1007420.s015.pdf]

S5 Table. Estimated parameter values.

Estimated parameters	Estimated value	Parameter bounds	Dimension	Respective reaction
k_1	5	1<br>-	h^{-1}	IFNAR degradation
k_2	19.9×10^{-6}	8.4×10^{-6}<br>41.4×10^{-6}	$(h \cdot \#)^{-1} \cdot nL$	IFNAR activation
k_3	11.5×10^{-2}	3.8×10^{-2}<br>23.0×10^{-2}	$(h \cdot \#)^{-1} \cdot nL$	IFNAR inactivation
k_4	0.1	-<br>0.3	h^{-1}	IFNLR degradation
k_5	5.5×10^{-5}	1.3×10^{-5}<br>24.4×10^{-5}	$(h \cdot \#)^{-1} \cdot nL$	IFNLR activation
k_6	10^{-6}	10^{-6}<br>1×10^{-3}	h^{-1}	IFNLR inactivation
k_7	2.6×10^{-4}	1.3×10^{-4}<br>3.9×10^{-4}	$(h \cdot \#)^{-1} \cdot nL$	STAT1/2 activation
k_9	0.6	0.3<br>0.9	h^{-1}	STAT1/2 inactivation
k_{10}	0.3	0.2<br>0.4	h^{-1}	ISG degradation
k_{11}	6.6	4.9<br>7.4	$(h \cdot \#)^{-1} \cdot nL$	ISG expression
k_{13}	0.4	0.2<br>0.8	h^{-1}	IFN degradation
$[IFNLR]_0$	250	100<br>400	$\# \cdot nL^{-1}$	Initial concentration of IFNLR

1- For fitting the high-affinity IFN-Lambda data, the IFNLR activation rate constant, k_5, was assumed five times larger. This assumption is based on the experimental observation that the modified IFN-lambda has five times higher affinity [32].
